# Description of patient reported experience measures (PREMs) for hospitalised patients with palliative care needs and their families, and how these map to noted areas of importance for quality care: A systematic review

**DOI:** 10.1177/02692163231169319

**Published:** 2023-04-24

**Authors:** Claudia Virdun, Maja Garcia, Jane L Phillips, Tim Luckett

**Affiliations:** 1 Faculty of Health, School of Nursing, Queensland University of Technology, Brisbane, Australia; 2 Faculty of Health, Improving Palliative, Aged and Chronic Care through Clinical Research and Translation (IMPACCT), University of Technology Sydney, Ultimo, NSW, Australia

**Keywords:** Palliative care, hospital, quality of care, quality improvement, surveys, questionnaires, systematic review

## Abstract

**Background:**

The global need for focused improvements in palliative care within the acute hospital setting is well noted. A large volume of evidence exists detailing what hospitalised patients with palliative care needs and their families note as important for high quality care. Patient Reported Experience Measures (PREMs) are one mechanism that hospitals could use to inform improvement work. To date there has not been a review of PREMs available for hospitalised patients with palliative care needs and/or their family, nor how they align with noted priorities for high quality care.

**Aim:**

To identify and describe PREMs designed for hospitalised patients with palliative care needs and their families; and their alignment with patient and family identified domains for high quality care.

**Design:**

A systematic review.

**Data sources:**

A systematic search of CINAHL, Medline and PsycInfo was conducted up to September 23, 2022 and supplemented by handsearching article reference lists and internet searches. PREMs written in English and designed for patients with palliative care needs in acute hospitals were eligible for inclusion. Included PREMs were described by: summarising key characteristics; and mapping their items to domains noted to be important to hospitalised patients with palliative care needs and their families informed by outcomes from a published study completed in 2021. Evidence for psychometric properties were reviewed.

**Results:**

Forty-four PREMs with 827 items were included. Items per PREM varied from 2 to 85 (median 25, IQR 13–42). Two-thirds (*n* = 534, 65%) of the items were designed for families and a third (*n* = 283, 34%) for hospitalised patients, and very few (*n* = 10, 1%) for both. Sixty-six percent of items measured person-centred care, 30% expert care and 4% environmental aspects of care. Available PREMs address between 1 and 11 of the 14 domains of importance for quality palliative care. PREMs had a median of 38% (IQR 25.4–56.3) of items >Grade 8 measured by the Flesch-Kincaid readability test, with Grade 8 or lower recommended to ensure health information is as accessible as possible across the population.

**Conclusions:**

Whilst 44 PREMs are available for hospitalised patients with palliative care needs or their families, a varied number of items are available for some domains of care provision that are important, compared to others. Few are suitable for people with lower levels of literacy or limited cognitive capacity due to illness.

**What is already known about the topic?**

- A substantial proportion of hospitalised patients are likely to be in the last 12 months of their life and therefore living with palliative care needs.
- The quality of palliative care for hospitalised patients is not routinely known at jurisdictional or national levels.
- Patient Reported Experience Measures (PREMs) are questionnaires that can be used to identify areas for improvement in health care provision from the perspectives of patients and families. However, it remains unclear which tools to use for appraising palliative care.

**What this paper adds**

- This review identified 44 PREMs available for use for hospitalised patients with palliative care needs and their families, and mapped them to domains of care shown to be important by previous research.
- This review identified variability in the number of items for some domains of care provision (e.g. communication and shared decision-making) that are important for patients and families, compared to other areas (e.g. cleanliness to support infection control).
- Many available PREMs contain items that are not suitable for people with lower levels of literacy or with limited cognitive capacity due to illness and its management.

**Implications for practice, theory or policy**

- Given the large numbers of PREMs available across the globe, it is important to firstly establish the core reason for use of a PREM so as to identify the optimal tool for use. Considering alignment with what matters most for patients with palliative care needs and their families is critical if improvement work is to be truly patient-centred.
- Mechanisms for data capture and timely feedback also need to be considered if these data are to inform the understanding of local needs, drive improvements and evaluate interventions.

## Introduction

Globally in high-income countries people are increasingly living longer with one or more non-communicable disease which will ultimately lead to their death.^1,2^ The leading causes of death by 2030 are projected to be: ischaemic heart disease, stroke, Alzheimer’s disease and other dementias, lung cancer, lower respiratory infections, chronic obstructive pulmonary disease, colorectal cancers, diabetes, hypertensive heart disease and kidney disease.^3^ All of these progressive and life limiting illnesses can inflict significant symptom burden, functional decline and inform a need for palliative care.

Currently, the majority of people dying from an expected death in a high-income country, do so within acute hospitals.^4–8^ In addition to people dying in hospitals, many people living with palliative care needs will require admissions within their last 12 months of life, with an estimated 27%–33% of a hospital population likely to have palliative care needs.^9–11^ These numbers are expected to rise^4,9,10,12^ and a growing proportion of admissions will be for end-of-life or terminal care, with an average length of stay of 10.6 days.^13^

Globally, acute hospitals in high-income countries struggle to ensure optimal palliative care for those who require it.^10,14–19^ Disjointed communication, too little input into decision making and poor symptom management all contribute to suboptimal care for hospitalised patients with palliative care needs.^5,15,20–22^ Sustained improvements in palliative care delivery within acute hospitals are challenging given the dominance of the biomedical model and its focus on cure,^19,22,23^ leading clinicians to provide a very problem-solution oriented approach to care rather than a proactive palliative approach.^15,24^

A recent study focused on characterising the domains of care that are most important to hospitalised patients with palliative care needs, and their families^25^ and the key drivers for enabling improvement in hospital palliative care delivery.^26^ In order to understand what hospitalised patients and families value for high-quality palliative care, patient and family perspectives were sought from international literature via a systematic review^27^ and metasynthesis.^28^ These studies revealed key domains of importance for quality care, informed predominantly by patients and families from high-income countries in the northern hemisphere. Adding to this work, a qualitative study informed by 21 hospitalised patients with palliative care needs and 29 families confirmed and added depth of understanding to these domains of care.^29^ Integrating these data sets, a meta-inference confirmed 14 domains of importance informed by data collected from 1233 hospitalised patients with palliative care needs and 3818 families.^25^ The 14 identified domains of importance confirm hospitalised patients and families require highly skilled, person-centred care that is provided within a therapeutic physical environment.^25^ Skilled care attributes important to hospitalised patients with palliative care needs and their families include excellence in physical care; impeccable assessment and care planning; effective symptom management; technical competence; patient safety; and supported access to senior clinicians.^25^ Person-centred care attributes have been described as including: respectful and compassionate care; effective communication and shared decision making; effective teamwork; enabling family involvement; and maintaining role, meaning and identity.^25^ Finally, both hospitalised patients and families identified that a high quality environment should include attention to cleanliness to support optimal infection control, alongside a range of other attributes that vary in importance between the two perspectives.^25^ This work also clarified the unique needs of hospitalised patients imminently dying and their families.^25^ These 14 domains of care are informed by thousands of perspectives from patients with palliative care needs and/or their families and therefore provide an ideal platform to guide focused improvement work.

Articulating key drivers to support clinical teams to progress this work was an important next step addressed via a co-design study designed to identify actions required to strengthen the delivery of palliative care in Australian hospitals so that it addressed the domains of care identified as important for hospitalised patients with palliative care needs and their families.^26^ This co-design study included 52 key Australian palliative care and acute hospital policy, consumer, medicine, nursing or allied health representatives and led to nine proposed actions to enable improvement including: (1) evidence-informed practice and national benchmarking; (2) funding reforms; (3) securing executive level support; (4) mandatory clinical and ancillary education; (5) fostering greater community awareness; (6) policy reviews of care of the dying; (7) better integration of advance care planning; (8) strengthen nursing leadership; and (9) develop communities of practice.^26^ One proposed action focused on the need for improved measurement to inform quality assurance and identify targets for improvement.^26^

Routine measurement of palliative care quality is not straightforward with many publications noting the potential role for patient reported outcome measures, patient reported experience measures, process measures and structural measures.^30–37^ More recent publications note the importance of prioritising the patient and family voice when possible to ensure care is aligned to what matters most from their perspectives.^30,33,35^ Patient reported experience measures (PREMS) are *‘. . .survey tools used to record patient perceptions about various elements of the healthcare they received*’^38^ (p.4) and are gaining prominence for their potential to identify areas for improvement in health care provision.^35,38,39^ There is a particular dearth of evidence regarding the routine use of PREMs to understand the quality of care for hospitalised patients with palliative care needs. Information is needed by clinical services on which PREM tools are available and best suited to appraising the quality of care against domains of importance to hospitalised patients living with palliative care needs and their families. Working from the domains of importance derived exclusively from hospitalised patient and family data^25^ ensures the identification of PREMs that measure what matters most to this population of patients and their families.

## Aim

To identify and describe PREMs designed for hospitalised patients with palliative care needs and their families; and their alignment with patient and family identified domains for high quality care.

## Methods

### Design

Systematic review and descriptive analysis of eligible PREMs and their measurement items. A systematic review of eligible PREM availability confers confidence in the state of play within this emerging field of practice, policy and research. A descriptive analysis of both the PREMs themselves and how they align to hospitalised patient and family identified areas of importance for quality care provides a useful platform from which to move forward. This systematic review has been reported in accordance with the PRISMA statement.^40^

### Operational definitions

Definitions informing this work include:

- Hospital – any metropolitan, rural or remote inpatient ward or unit excluding psychiatric, hospice or inpatient specialist palliative care, and alcohol and drug treatment centres.
- Hospitalised patients with palliative care needs and their families – adult patients (aged 18 years or over) predicted to be in the last 12 months of their life, informed by having one or multiple life-limiting conditions in accordance with the Supportive and Palliative Care Indicators Tool (SPICT ™).^41^ Families were those identified by the person as family inclusive of those biologically related, members of the community and others the patient agrees to being involved in their care.^42^

### Eligibility criteria

Eligibility pertained to PREMs, rather than articles. Eligible PREMs were those written in English and designed to capture the experiences of hospitalised patients with palliative care needs and/or their families of care received in hospital settings. PREMs did not have to be validated and/or published in the peer-review literature to be included. Items or PREMs that were overall ratings of global quality of care were deemed too general to be informative to quality improvement and so excluded from this review.

**Textbox.** Inclusion and exclusion criteria for PREMs.

Inclusion criteria	Exclusion criteria
Written in English	Designed for paediatrics
Designed for patients with palliative care needs and/or their families	Designed for the community or aged care setting
Designed for adults	Written in a language other than English without a translation readily available
Designed for use in the hospital setting	Focused on global ratings of quality only
	Focused specifically on one diagnostic group (e.g. a PREM designed for people with Heart Failure rather than for the broader population of people with palliative care needs)

### Information sources

An initial search was conducted on 19th November 2021 of CINAHL (EBSCO Host) and Medline (OVID), with the addition of PsycInfo (EBSCO Host) because of its focus on psychometric studies. This search was updated on 23 September 2022 to identify any further citations. Searches of the internet via Google and Google Scholar search engines and the Australian online palliative care knowledge network CareSearch were also completed. The reference lists of all included studies and other relevant reviews were searched manually to identify other potentially relevant articles. Given the relevance of the recent systematic review^27^ and metasynthesis^28^ completed by members of this research team to identify key domains of importance for optimal palliative care for hospitalised patients, forward citations from these two publications were reviewed.

### Search strategy

Previous systematic reviews informed the development of search terms for palliative care, patient, family or family members,^28^ and for terms encompassing patient experience and satisfaction questionnaires and questionnaire terms^39^ (Refer to Table 1). See Supplemental Tables 1–3 for full search strategies used per database.

**Table 1.** Search terms used – Palliative care PREMs.

1. dying, death, ‘end of life’, terminal, ‘terminal care’, terminally ill, palliative, ‘final day*’ (combine all with ‘or’) 2. ‘good death’, ‘consumer satisfaction’, ‘patient satisfaction’, perspective*, important, experience (combine all with ‘or’) 3. Hospital, acute care, intensive care, emergency, inpatient* (combine all with ‘or’) 4. Patient*, family, families, consumer*, family* (combine all with ‘or’) 5. Adult* 6. (Patient* or Consumer*) adj (satisfaction or experience* or opinion* or perspective*) and (questionnaire* or instrument* or measure) or Patient Reported Outcome Measures **7. 1 and 2 and 3 and 4 and 5 and 6** **8. Limit ‘7’ with 1990 – current and English language** ***9. NB: Slight variations with truncations were used to account for database requirements***

### Selection

The process for screening and inclusion were determined a priori and included:

1. Initial abstract and title searching of whether a paper referred to a PREM developed for hospitalised patients with palliative care needs (completed by one reviewer – MG – an experienced research assistant with content knowledge). This was determined to be appropriate given the criteria were objective and clear;
2. Once papers with PREMs for people with palliative care needs were identified, these were reviewed by two researchers (MG and TL) to finalise inclusion/exclusion. If any uncertainty arose, a consensus discussion was held with the full research team. Areas of uncertainty tended to be either in relation to whether a survey tool was indeed a PREM, or a mix of items (both outcome and experience measures); and/or whether it had been designed for use by hospitalised patients with palliative care needs. Discussions with the broader research team (CV, MG, TL and JP) resolved these uncertainties given experience and skill-mix in relation to PREMs, other survey tools and palliative care;
3. MG searched for the PREM tools referred to within the included papers and extracted all measurement items. Inclusion and exclusion of items was determined by three researchers (MG, TL and CV) with consensus discussions held as needed with the full research team.

### Data extraction

Eligible PREMs were entered into a summary table. Data extracted for each PREM included: the date of when the PREM was published; the PREM name, the number of items, its country of origin, who developed it, a general overview of the purpose of the PREM and who the PREM was designed to be administered to (patient; family or both). PREM items were then extracted from each PREM using an MS Excel proforma for further analysis and mapping to domains of importance. Items were defined as the questions in PREMs designed to elicit a response from participants. Only closed-ended items were extracted from PREMs. Furthermore, data informing quality appraisal were also extracted into an Excel spreadsheet for analysis.

### Quality appraisal

The evidence for psychometric properties of each PREM was appraised based on criteria outlined by COnsensus-based Standards for the selection of health Measurement INstruments (COSMIN), including validity, reliability and responsiveness.^43^ Interpretability was considered less relevant for PREMs than patient-reported outcome measures because it focuses on evidence for minimal clinically important differences and normative data on distribution of scores from large representative datasets for comparison purposes. Aspects of reliability that were appraised included test-retest, inter-rater, intra-rater, measurement error and internal consistency. Aspects of validity included content validity, face validity and construct validity (structural, cross-cultural and hypothesis testing). Criterion validity was not considered relevant for PREMs since no gold standard exists for appraising quality of palliative care. Evidence for psychometric properties was appraised by one reviewer (MG), with discussions with others as needed to reach consensus.

### Synthesis

All PREM items were independently mapped by two reviewers (MG and CV) to the domains of importance for optimal palliative care for hospitalised patients^25,27,28^ using MS Excel. Mapping was informed by the key practice points aligned to each domain^25^ and further refined through discussion with the team. MG collated the two mappings and colour coded these as green (agreement) and red (disagreement). All red coded items were then discussed with a third reviewed (TL) until consensus was reached. Items were also assessed by the Flesch-Kincaid et al. readability test^44^ to compare reading grades of included items, to acknowledge the cognitive limitations often associated with hospitalised patients with palliative care needs. The ideal reading level for health information should be at grade eight or lower to ensure that the content is accessible to people with lower levels of education.^45^ This is particularly important for people with palliative care needs who are noted to have high levels of potential cognitive impairment due to advanced serious illness and/or treatment related effects.^46^

## Results

**Figure 1.**
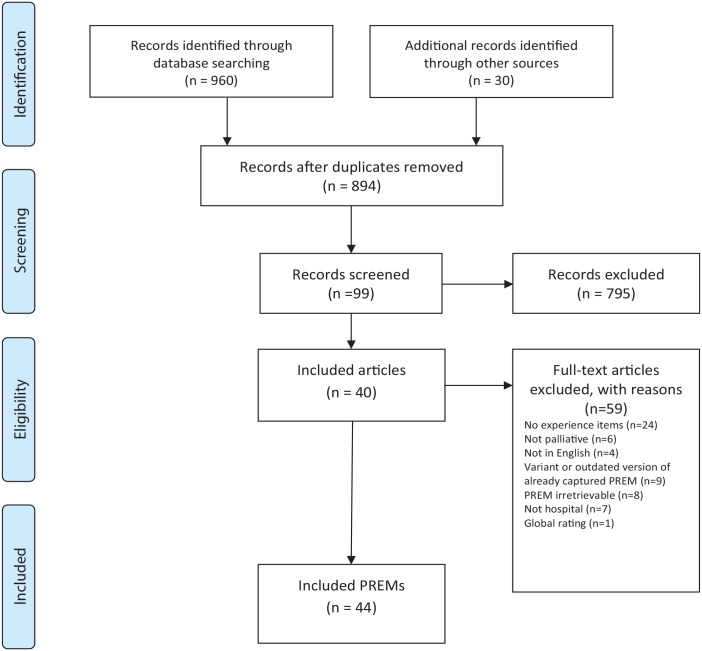
PRISMA flowchart showing selection process for included patient/family experience PREMs.

The search retrieved 960 citations: MEDLINE (*n* = 653); CINAHL (*n* = 179) and PsycInfo (*n* = 128), and 30 citations were identified through hand-searching grey literature. De-duplication left 894 citations (see Figure 1). After screening the title and abstracts, 99 publications reporting on patient/family PREMs were identified. Fifty-nine publications were excluded as they were: not in English; not concerned with patient/family experience; not developed for use with a palliative population; developed for a paediatric population; were focused only on symptom assessment or quality of life without questions relating to patient experience; or were reporting on a modified or outdated version of an already retrieved PREM (see Figure 1). Five of these PREMs were different variants of the PREM CANHELP (CANHELP Patient, CANHELP Caregiver, CANHELP Bereavement; CANHELP Lite Patient and CANHELP Lite Caregiver).^47–49^ Due to the similarity in wording, CANHELP Bereavement and CANHELP Caregiver^47^ was considered one PREM, and the Lite versions^49^ were excluded in analysis to minimise duplication of items. This left 40 publications reporting on the use of 44 PREMs to be mapped across the domains of areas of importance for optimal palliative care provision in the hospital setting. A summary of PREMs is provided within Table 2 (patient PREMs) and Table 3 (family PREMs). An updated search run in September 2022 retrieved 43 citations that were reviewed with none meeting stated inclusion criteria.

**Table 2.** Characteristics of included PREMs for patients receiving palliative care.

PREM name, date of publication presented in chronological order, country	Purpose	No. of items	% of items above Flesch-Kincaid grade 8
consideRATE, 2021^50^* USA	A measure of serious illness experience based on what matters most to people who are seriously ill.	8	0
Palliative Care Clinical Network – Palliative Care Experience Survey,^51^ 2020* Australia	An online survey to collect information on experiences of palliative care quality in South Australia from perspectives of patients, families or health professionals.	14	14.3
The Sinclair Compassion Questionnaire (SCQ), 2020^52^ Canada	Evaluates compassion as perceived by patients in the care they received from a facility.	15	33.3
Quality Care Questionnaire-Palliative Care (QCQ-PC), 2018^53^ Korea	Evaluates four factors: communication with health care professionals; discussing value of life and goals of care; support and counselling for holistic care needs; and accessibility and continuity of care in patients receiving palliative care.	32	46.9
Victorian Palliative Care Satisfaction Instrument (VPCSI),^54^ 2016* Australia	Assesses patient and family satisfaction with palliative care provision from services across Victoria, Australia.	58	74.1
Feeling Heard and Understood, 2015^55^ USA	The Heard and Understood measure was developed for patients with advanced cancer who receive inpatient palliative care consultation, to measure the degree to which they feel heard and understood by those caring for them in the hospital environment.	2	50
Quality from the Patient’s Perspective (QPP-PC), 2015^56^ Norway	Measures the quality of palliative care from patients’ perspectives across a variety of care contexts.	51	33.3
Patient Satisfaction Questionnaire, 2014^57^ United Kingdom	Evaluates doctor’s communication and interpersonal skills, from the perspective of patients with palliative care needs in the inpatient hospice setting.	12	33.3
Quality of End-of-life Care and Satisfaction with Treatment (QUEST)^58^ questionnaire, 2013 USA	This questionnaire allows dying patients to rate the quality of care they received from doctors and nurses, and their satisfaction with care.	15	40.0
Satisfaction with treatment decision (SWTD) survey, 2013^59^ Switzerland	Measures the success of a patient-doctor encounter during consults where new goals of treatment are identified, and satisfaction with subsequent decisions in patients receiving a new line of palliative treatment.	6	66.7
Consumer Quality Index Palliative Care questionnaire for patients, 2012, 2013^60^ The Netherlands	Assesses the quality of palliative care from the perspective of patients to give health professionals insight into which aspects of care to prioritise in quality improvement.	32	40.6
Satisfaction with Doctors Questionnaire, 2011^61^ Turkey	A self-administered questionnaire to determine the expectations and satisfaction levels of Turkish patients receiving palliative care with their doctors and their preferences about death.	8	12.5
Quality of End-of-Life Care (QOELC) Survey – Patient, 2010^62^ USA	The QEOLC is an instrument in which patients with palliative care needs rate a clinician’s skill at providing high quality end-of-life care.	11	54.5
FAMCARE-Patient, 2009^63^ Australia	A self-report scale initially designed to assess patient satisfaction in outpatient palliative oncology care.	13	61.5
Quality of Communication Questionnaire (QOC), 2006^64^ USA	A 13-item patient-centred, patient-report questionnaire about the quality of end-of-life communication.	13	38.5
Palliative Care Quality of Life Instrument (PQLI), 2004^65^ Cyprus	The PQLI was created specifically to capture perspectives of patients with advanced cancer receiving palliative care on their quality of life.	28	7.1

* PREM was aimed at both patients and families.

**Table 3.** Characteristics of included PREMs for families of patients with palliative care needs.

PREM name, date of publication presented in chronological order, country	Purpose	No. of items	% of items above Flesch-Kincaid grade 8
Kaiser Permanente Survey, 2020, 2001^66^ USA	To understand how well the medical system at Kaiser Permanente South California was delivering end of life care.	46	23.9
Marti-Garcia et al. 2020 study developed questionnaire^67^* Spain	To gather the perceptions of patients and their caregivers on the experience of end-of-life care in varied clinical contexts.	74	35.1
Otani et al. 2020 study developed questionnaire^68^ Japan	A nationwide survey of bereaved family members of people with cancer, aimed at evaluating quality of end-of-life care across Japan.	12	8.3
Palliative Care Clinical Network – Palliative Care experience Survey, 2020^51^* Australia	An online survey to collect information on experiences of palliative care quality in South Australia from perspectives of patients, families or health professionals.	14	14.3
Care of the Dying Evaluation (CODE)/international CODE (i-CODE), 2019^69^ United Kingdom	A post-bereavement questionnaire focused on both quality of patient care and the level of family-family support provided in the last days of life and immediate post-bereavement period.	42	28.6
Dying Care Outcome and Process Scale Before and After Death, 2019^70^ Japan	Evaluates the process and outcomes of care during the last days of life and after death based on bereaved family members’ perspectives.	8	0
VOICES-SF (short form), 2019^71^ United Kingdom	A shortened version of the postal ‘Views of Informal Carers – Evaluation of Services’ questionnaire, which collects information on bereaved people’s views of the quality of care provided to a friend or relative in the last 3 months of their life.	59	16.9
Consumer Assessment of Healthcare Providers and Systems (CAHPS) Cancer Care Survey, 2017^72^ USA	Assesses the experiences of adult patients with cancer treatment provided in outpatient and inpatient settings.	85	7.1
Caregiver Voice Survey, 2017^73^ Canada	Investigates the experience of end-of-life care for patients and their caregivers.	62	29.0
euroQ2 Satisfaction with Care in the ICU, 2017^74^ Denmark	To capture the experiences of families in ICU. This is an adaptation of the ‘Quality of Dying and Death questionnaire’ and the ‘European Family Satisfaction in the ICU’.	33	24.2
Bereaved Family Survey (BFS), 2016^75^ USA	Evaluates the quality of care and outcomes of end-of-life care for veterans dying in inpatient Veterans’ Affairs facilities across the USA and Puerto Rico.	17	35.3
Victorian Palliative Care Satisfaction Instrument, 2016^54^* Australia	Assesses the satisfaction of patients and families with palliative care provision from services across Victoria, Australia.	58	74.1
Family Evaluation of Hospice Care (FEHC), 2015^76^ USA	One of the most widely used instruments employed by U.S. hospices to evaluate family perspectives of hospice programmes.	61	38.9
Quality of Family Experience (QUAL-E Fam), 2014^77^ USA	Measure of the quality of palliative care experience for family members of patients who are terminally ill.	17	100
Consumer Quality Index Palliative Care questionnaire for relatives, 2013^60^ The Netherlands	To assess the quality of palliative care from the perspective of bereaved relatives.	22	68.2
Canadian Health Care Evaluation Project (CANHELP) – Bereavement/Caregiver, 2010^47^ Canada	Evaluates satisfaction with care for older patients with life threatening illnesses, and their family members.	40	70.0
CANHELP Patient, 2010^47^ Canada	Evaluates the satisfaction with care for older patients with life threatening illnesses and their family members.	38	78.9
FAMCARE-2, 2010^78^ Australia	Measures satisfaction of family members with palliative care received by patients and their family members.	17	47.1
Quality of End-of-Life Care (QOELC) Survey – Family, 2010^62^ USA	Evaluates a clinician’s skill at providing high quality end-of-life care from the bereaved family’s perspective.	22	50.0
Family Assessment of Treatment at the End-of-Life (FATE), 2008^79^ USA	Assesses the quality of end-of-life care in a population, regardless of setting. FATE items evaluate respondents’ perceptions of outcomes of care by asking them to assess how well the care provided met the needs of the patient and his/her family.	32	62.5
Family Satisfaction with Care in the Intensive Care Unit: FS-ICU 24R, 2007^80^ Canada	Measures family satisfaction with the care given to the patient and their family during an ICU admission.	30	16.7
CAHPS Hospice Survey, 2006^81^ USA	Assesses the experiences of patients who died while receiving hospice care, and the experiences of their primary informal caregivers.	47	29.8
Family Evaluation of Palliative Care (FEPC), 2005^82^ USA	Captures family members’ perceptions about the quality of palliative care provided—whether that care was provided by a hospital-based consult service or by a hospice programme offering palliative care.	45	42.2
Care Evaluation Scale (CES), 2004^83^ Japan	Measures the bereaved family’s perception about necessity for improvement in structure/process aspects of palliative care.	28	64.3
Quality of Death and Dying Questionnaire (QODD), 2002^84^ USA	Interviewer-administered PREM asking bereaved families to rate the quality of the dying experience for the decedent’s last 7 days or, if the patient was unconscious or unresponsive during the last 7 days, over the last month before death.	31	25.8
Satisfaction Scale for Family Members Receiving Inpatient Palliative Care (SFIPC), 2002^85^ Japan	Measures informal family satisfaction with inpatient palliative care.	60	35.0
End-of-Life in Dementia Satisfaction With Care (SWC-EOLD), 2001^86^ USA	Evaluates the experience of end of life care for severely cognitively impaired persons from the perspectives of bereaved families.	10	40.0
After death bereaved family member interview (hospital version) (Part of the Toolkit of Instruments to Measure End of Life Care [TIME]), 2000^87^ USA	Examines whether end of life care meets the expectations and needs of the dying person and their family members.	42	88.1
Primary Caregivers Satisfaction Survey, 1999^88^ USA	Assesses the degree of satisfaction of families of patients receiving care from hospices with support received from social work staff.	12	100

* PREM was aimed at both patients and families.

### Quality appraisal

Supplementary Table 4 summarises evidence available for psychometric properties for each PREM, as defined by COSMIN. The following PREMs had no evidence available for any psychometric property and have been excluded from the table: Palliative Care Clinical Network (PCCN) Experience Survey,^51^ Feeling Heard and Understood,^55^ Patient Satisfaction Questionnaire,^57^ Satisfaction with Doctors Questionnaire,^61^ Marti-Garcia et al. 2020 study developed questionnaire,^67^ VOICES-SF,^71^ Victorian Palliative Care Satisfaction Instrument (VPCSI),^54^ and the Family Evaluation of Palliative Care (FEPC).^82^ The euroQ2^74^ includes two PREMs that have been individually validated, but has not been validated as a single questionnaire. Of remaining PREMs, the Caregiver Voice Survey^73^ was the one with evidence available for the highest number of psychometric properties, lacking evidence only for responsiveness. Indeed, responsiveness – along with cross-cultural validation – was the property that was least tested among PREMs.

Of the 44 PREMs, most were generated in the USA (*n* = 18)^50,55,58,62,64,66,72,75–77,79,81,82,84,86–88^with smaller numbers emerging from Japan (*n* = 4) ^68,70,83,85^; Australia (*n* = 4) ^51,54,63,89^; the UK (*n* = 3) ^57,69,71^; and the Netherlands (*n* = 2).^60,90^ The remainder were from other high-income countries (*n* = 11)^48,49,52,53,56,59,65,67,73,74,80^ or Turkey (*n* = 1).^61^ Of the 44 PREMs, three quarters (*n* = 34, 77%) had some level of data available about psychometric properties. Items within each PREM varied from 2 to 85, median number of items 25, (IQR 13–42). Across all PREMs, the median proportion of items that scored above grade eight on the Flesch Kincaid test was 38% (range 0%–100%). In two PREMs all items^77,88^ scored above grade eight, whilst another two contained no items that scored above grade 8.^50,70^ Included PREMs were developed for administration to patients (*n* = 15),^47,52,53,55–65,84^ families (*n* = 26)^47,60,62,66–77,79–83,85–89^ or both (*n* = 3).^50,51,54^

**Figure 2.**
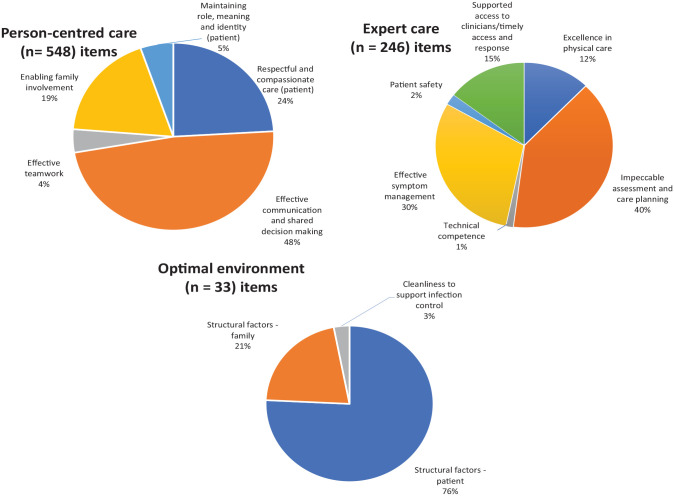
Proportion of all items (patient and family focused) mapped across domains of importance.

From the 44 PREMs, 827 items assessing patient or family experience of care were available for analysis. More than half of the items were designed for use with families compared to patients (*n* = 534; 65% vs *n* = 283; 34%). Only a very small number of items (*n* = 10; 1%) assess both patient and family experiences. The number of domains addressed by each PREM ranged from 1 to 11, with the CANHELP PREM (both patient and caregiver versions), addressing the most domains (*n* = 11) (Table 4 with additional details in the online Supplemental File). Figure 2 shows the proportions of items addressing the domains of importance.

**Table 4.** PREM items mapped against domains of importance.

	Respectful and compassionate care	Effective communication	Effective teamwork	Enabling family involvement	Maintaining role, meaning and identity	Excellence in physical care	Impeccable assessment and care planning	Technical competence	Effective symptom management	Patient safety	Supported access to clinicians	Structural factors – patient	Structural factors – family	Cleanliness	Total number of domains addressed
CANHELP (Caregiver)	6	11	1	4	2	1	2	0	1	1	2	1	0	0	11
CANHELP (Patient)	6	11	1	1	7	1	2	0	1	1	2	1	0	0	11
CQIPC (Patient)	6	7	5	1	2	3	2	0	4	0	1	1	0	0	10
CES	5	8	2	2	1	1	2	0	1	0	0	3	0	0	9
QPP-C	3	10	0	2	2	4	5	0	7	0	4	2	0	0	9
CaregiverVoice	3	6	0	2	2	2	6	0	6	0	0	1	0	0	8
CODE	4	9	0	3	0	2	2	0	3	0	0	2	0	1	8
FATE Survey	2	12	0	4	0	1	2	0	0	1	1	1	0	0	8
FEPC	2	11	1	6	0	1	2	0	3	0	2	0	0	0	8
QCQ-PC	2	15	0	2	1	0	6	0	1	0	1	1	0	0	8
SFIPC	5	8	0	4	0	1	1	0	3	0	1	0	7	0	8
TIME	4	17	2	3	0	4	4	0	6	0	3	0	0	0	8
VPCSI	6	8	1	21	0	0	0	1	4	0	6	9	0	0	8
VOICES-SF	3	4	1	4	0	2	2	0	1	0	0	1	0	0	8
FAMCARE-2	1	3	0	2	0	3	3	0	4	0	1	0	0	0	7
CQIPC (Relatives)	5	8	2	3	0	0	2	0	0	0	1	0	0	0	6
EOLD-SWC	0	4	1	1	0	1	2	0	0	0	1	0	0	0	6
FAMCARE (Patient)	0	6	1	0	0	1	1	0	3	0	1	0	0	0	6
QOELC (Patient)	1	3	1	1	0	0	4	0	0	0	1	0	0	0	6
QODD	1	6	0	0	2	0	5	0	2	0	3	0	0	0	6
CAHPS	2	6	0	4	0	0	4	0	3	0	0	0	0	0	5
Considerate	1	2	0	0	0	0	2	0	1	0	0	1	0	0	5
FEHC	1	6	0	7	0	1	0	0	1	0	0	0	0	0	5
Marti-Garcia et al. 2020	0	19	0	8	0	0	5	0	5	0	1	0	0	0	5
QOELC (Family)	4	8	0	1	0	0	6	0	0	0	2	0	0	0	5
BFS	1	4	0	0	0	1	3	0	0	0	0	0	0	0	4
CAHPS Cancer Care Survey	3	4	1	0	0	0	1	0	0	0	0	0	0	0	4
DPS-B	2	1	0	4	0	0	0	0	1	0	0	0	0	0	4
euroQ2	1	3	0	0	0	0	5	0	3	0	0	0	0	0	4
Archer et al. 1999	1	2	0	8	0	0	1	0	0	0	0	0	0	0	4
QOC	3	8	0	0	1	0	1	0	0	0	0	0	0	0	4
QUEST	22	2	0	0	0	0	0	2	0	0	2	0	0	0	4
DOS-B	3	0	0	3	0	0	0	0	1	0	0	0	0	0	3
FS-ICU 24 R	2	0	0	0	0	0	8	0	5	0	0	0	0	0	3
KPS	0	6	0	0	0	0	4	0	3	0	0	0	0	0	3
Otani et al. 2020	0	0	0	0	9	0	0	0	0	2	0	1	0	0	3
SCQ	10	2	0	0	0	0	3	0	0	0	0	0	0	0	3
PCCN Consumer/ Carer Survey	0	2	0	0	0	0	0	0	1	0	0	0	0	0	2
PQLI	3	0	2	0	0	0	0	0	0	0	0	0	0	0	2
PSQ	5	6	0	0	0	0	0	0	0	0	0	0	0	0	2
Durusoy et al. 2011	3	4	0	0	0	0	0	0	0	0	0	0	0	0	2
FHU	0	2	0	0	0	0	0	0	0	0	0	0	0	0	1
QUAL-E (FAM)	0	4	0	0	0	0	0	0	0	0	0	0	0	0	1
SWDS	0	6	0	0	0	0	0	0	0	0	0	0	0	0	1
Total items	132	264	22	101	29	30	98	3	74	5	36	25	7	1	

BFS: Bereaved Family Survey; CAHPS: Consumer Assessment of Health Providers and Systems; CES: Care Evaluation Scale; CODE: Care of Dying Evaluation; CQIPC: Consumer Quality Index Palliative Care; DOS: Dying Outcome Scale; DPS: Dying Process Scale; EOLD-SWC: Satisfaction with Care at the End of Life in Dementia; euroQ2: Satisfaction with Care in the ICU; FAMCARE: Family Satisfaction with Care; FATE: Family Assessment of Treatment at the End of Life; FEHC: Family Evaluation of Hospice Care; FEPC: Family Evaluation of Palliative Care; FHU: Feeling Heard and Understood; F-ICU 24R: Family Satisfaction with Care in the Intensive Care Unit; KPS: Kaiser Permanente Questionnaire; PCCN: Palliative Care Clinical Network; PCQLI: Palliative Care Quality of Life Instrument; PSQ: Patient Satisfaction Questionnaire; QCQ-PC: Quality Care Questionnaire Palliative Care; QOC: Quality of Communication Questionnaire; QODD: Quality of Death and Dying Questionnaire; QOELC: Quality of End of Life Care; QPP-PC: Quality from the Patient’s Perspective for Palliative Care; QUAL-E-FAM Quality of Family Experience; QUEST: Quality of End of life Care and Satisfaction with Treatment; SCQ: Sinclair Compassion Questionnaire; SFIPC: Satisfaction Scale for Family Members Receiving Inpatient Palliative Care; SWDS: Satisfaction With Decision Scale; TIME: Toolkit of Instruments to Measure End of Life Care; VOICES: Views Of Informal Carers—Evaluation of Services; VPCSI: Victorian Palliative Care Satisfaction Instrument.

## Person-centred care (number of items = 548)

### Respectful and compassionate care (number of items = 132)

Of the 132 items extracted from 35 questionnaires that assessed ‘respectful and compassionate care’ more than half (*n* = 70) targetted patients, 61 items were specifically for families, and one was designed for the use of either or both patient and family. Items were crafted to determine if patients were treated with ‘dignity and respect’, or to ask patients or their families about trust and confidence in their clinicians.

### Effective communication and shared decision making (n = 264)

Of the 264 items extracted from 40 PREMs that assessed ‘effective communication and shared decision making’ more than half (*n* = 169) sought families feedback, 91 items were aimed at patients, and four were aimed at either patients or families. These items tended to ask about whether patients or families felt they were listened to, whether patients and/or families wishes or preferences for care were considered or whether patients and/or families felt they were given sufficient information to make decisions.

### Effective teamwork (n = 22)

Of the 22 items extracted from 14 PREMs that assessed ‘effective teamwork’ half (*n* = 11) were aimed at families, with the other half aimed at patients. These items tended to ask if respondents could identify who oversaw the patients’ care, or if they received consistent or contradictory information between different health professionals.

### Enabling family involvement (n = 101)

Of the 101 items extracted from 26 PREMs that assessed ‘enabling family involvement’, 93 items specifically targetted families and a smaller number sought patients (*n* = 8) feedback. While these items tended to ask if families were supported emotionally, or whether they were supported or felt confident that they can care for the patient at home, some items also sought to determine the emotional or practical support families received following the patient’s death.

### Maintaining role, meaning and identity (n = 29)

There were 29 items identified from 10 PREMs that assessed ‘maintaining role, meaning and identity’. Of these, 14 items were aimed at families, and 15 were aimed at patients. These items tended to ask patients if they were supported outside of their illnesses, such as if they felt that they can discuss economic or social problems with their doctors, or whether their nurses asked about their everyday habits to help them maintain their daily lives.

## Expert care (number of items = 246)

### Excellence in physical care (n = 30)

Excellence in physical care was assessed in 30 items across 14 PREMs. Of these items, 21 were aimed at families and 9 at patients. These items tended to ask respondents about whether personal needs, such as washing or toileting needs, or assistance with positioning in bed were met.

### Impeccable assessment and care planning (n = 98)

There were 98 items identified from 31 PREMs that assessed impeccable assessment and care planning. Of these items, 67 were aimed at families and 29 at patients. Items mapped to this domain tended to be about whether families felt that they knew what to expect about their relatives’ conditions, including if they expected their relatives’ death. A number of items assessing whether or not patients’ emotional or spiritual needs were met were also mapped to this domain.

### Technical competence (n = 3)

There were three items identified that assessed technical competence across two PREMs. Four items were aimed at families and six at patients. Items in this domain tended to ask patients and families whether or not they felt that their doctors or nurses had the knowledge or technical skills to manage their care.

### Effective symptom management (n = 74)

There were 74 items identified from 26 PREMs that assessed effective symptom management. Of these items, 54 were aimed at families, 18 at patients and two at both patients and families. Items mapped under this domain asked patients if their symptoms were sufficiently managed and whether families felt sufficiently supported in managing symptoms of their relatives.

### Patient safety (n = 5)

Only five items assessed patient safety, from four PREMs. Four of these items were aimed at families, asking families if they felt satisfied that their relatives received good care when families were not present, or whether families felt that their relatives felt safe and assured.

### Supported access to senior clinicians (n = 36)

There were 36 items identified from 19 PREMs that assessed ‘supported access to senior clinicians’. Of these items, 20 were aimed at families and 16 were aimed at patients. Most items tended to consider access to clinicians in relationship to timely response of nurses in the hospital setting and access to specialists. Items also evaluated the timeliness of access to clinicians during acute situations or crises out of hours at the hospital.

## Optimal environment (number of items = 33)

### Structural factors – patient (n = 25)

There were 25 items identified from 13 PREMs that assessed ‘structural factors’ that impacted on the patient care experience. Of these items, 12 were aimed at assessing the families’ perspective, and 12 were aimed at patients and one was aimed at both patients and families. These items tended to ask if patients and families felt that their environments in hospital were comfortable, had adequate privacy, enabled access to high quality food or visitation of family members/friends.

### Structural factors – family (n = 7)

There were seven items from one PREM that assessed ‘structural factors’ that impacted on families’ experiences. All items were aimed at families. These items asked if families found the facilities to be adequately private, whether or not they received appropriate refreshments or whether they had access to a function or family room.

### Cleanliness to support infection control (n = 1)

Only one item assessed ‘cleanliness to support infection control’, which broadly asked families if they were satisfied with the cleanliness of their relative’s environment.

### Items that did not directly meet the domains of importance

A small number of items did not directly address the domains of importance but were mapped to the domain that was the most closely representative of what they were assessing. Items that attempted to measure the extent of the burden of care on families (*n* = 3) or the need for financial support (*n* = 1) were mapped to ‘Enabling family involvement’. A small number of items addressing cultural and spiritual needs (*n* = 4) were mapped to ‘Impeccable assessment and care planning’.

## Discussion

Of the 44 PREMs that included items for measuring care identified to be important by hospitalised patients with palliative care needs and/or their families, most focused on families’ experience. This reflects established barriers to obtaining self-reports from palliative patients who may be experiencing significant symptom burden, cognitive overload or impairment and in the end stages of disease with lower levels of consciousness.^46,91^ Importantly this review highlights that a large proportion of items are written at a level of English deemed to be too complex for a large proportion of the population^45^ without taking into account that many have fluctuating cognitive capabilities related to their acute illness (i.e. delirium) or medications.^46^

There is an uneven distribution of items across domains of importance, ranging from 741 available items for ‘effective communication and shared decision-making’ down to only one for ‘cleanliness to support infection control’.^69^ The relative attention to different domains by PREMs developers does not reflect how patients and families have weighted these domains in terms of importance, but rather what might be perceived as easier to collect or of greater clinical importance, as defined by health professionals or organisations. This variance is important as the domains least addressed within current PREMs include: effective teamwork; maintaining role, meaning and identity, excellence in physical care, technical competence, patient safety, supported access to senior clinicians and therapeutic environmental factors. All of these domains, except maintaining role, meaning and identity are noted to be important from both the patient and family perspective.^25,27–29^ Patients uniquely note the importance of maintaining role, meaning and identity specifically noting the importance of them feeling supported and encouraged to maintain as much independence as possible within their individual context, to engage in meaningful activity on a daily basis and to feel a sense of control within the institutionalised environment of the hospital setting.^25^ This study informs that less than 5% of available items map to these noted areas of importance. In taking this work forward, it is important that PREMs align to what matters from a patient and family perspective, especially if these measures are to be used to improve care outcomes.^25,27–29^

Finding the right balance between domains of importance and brevity is an additional important consideration for unwell patients with palliative care needs.^46^ The PREMs identified by this review ranged from two questions through to 85, with an average of 30 questions across all PREMs. This is likely to be too long and burdensome for patients with palliative care needs.^50^ In response to this challenge, a team from the United States leveraged earlier work completed by members of this research team, detailing care that matters most for hospitalised patients with palliative care needs and their families,^27^ to co-design a brief PREM.^50^ This eight item ConsideRATE PREM^50,92^ aligns closely to some of the key areas of importance whilst not being comprehensive across all domains of importance,^25,27,28^ to prioritise brevity. The PREM concludes with an open ended question to enable patients to share any additional feedback not captured within the presented questions.^50^ Initial ConsideRATE validation work has been completed, with preliminary evidence suggesting that it is acceptable and easy to use.^50,92^ While it purports to measure both patient and family experience, the PREM’s items focus on care for the patient rather than issues more relevant to families. Validation of proxy ratings for the use of this PREM are not available.

### Future areas of research

Four key areas of research have been highlighted in this review to progress the successful embedding of the use of PREM data into routine practice, namely: (1) how to embed PREMs into practice with a close review of implementation factors within the acute care setting – what tool, what mechanisms to support use, what is feasible and acceptable?; (2) understanding the impact of PREM measurement and reporting on patient and family outcomes and how to best evaluate this?; (3) understanding whether and how PREM data drives actionable change at micro (ward level), meso (organisational level) and macro (jurisdictional and national levels) and how these changes intersect with improved patient, family and clinical experiences; and (4) ongoing psychometric analysis to inform the understanding of how robust measures are with a particular view to better understand the impact of proxy ratings and how to best manage the inherent bias within measurement of experience.

Variance in how patients with palliative care needs, and their families want to contribute to and engage with quality improvement is noted.^35,93^ Some suggest that the use of electronic devices for data capture is acceptable while others do not.^93^ In addition, the ability for PREMs to directly inform care provision to individuals through integration with real-time care planning and provision is hampered by the fact a proportion of patients and families want to remain de-identified when providing a rating for fear of retribution if they are critical of care.^50,93^ A-priori decisions are therefore needed in relation to how these PREMs will be used, what they seek to inform (current care or service improvement work) and how the evaluation of their impact is measured. Positioning all such decision making within the busy and chaotic contexts of hospitals is another critical factor for careful consideration.^93^ The complexity of noting the ideal PREM and ideal implementation factors for people with palliative care needs is clear. However, research notes the need to prioritise brevity due to patient factors (fatigue and cognition)^46,50,92^ and system factors (busy clinical settings).^50,92,93^ This review provides a foundation upon which to progress this work through a systematic review of current PREMs available and how they align to areas of importance for high quality care from the perspectives of patients and families. Understanding how to implement such tools into routine practice remains an important area for focus.

### Strengths and limitations

The strength of this review lies in the systematic methodology used to locate PREMs, limit bias and assimilate large amounts of information to inform future health service planning. The linkage of available PREM items to noted areas of importance for patients and families ensures study outcomes reflect what matters for people with palliative care needs. However, there are also limitations. Firstly, a single author (MG) performed the initial screening of titles and abstracts, located PREMs and led the data extraction. This same author (MG) performed the appraisal of evidence for psychometric properties under careful guidance from TL. However, where uncertainty existed, discussion with the research team was undertaken for a consensus view. Secondly, the theming to areas of importance was subjective given the fact some items could be mapped to multiple domains. Therefore, consensus decisions were made to map to the domain that was most relevant. Finally, the domains of importance noted within this review are not reflective of culturally and linguistically diverse or Indigenous populations and this needs to be considered when planning implementation at system levels.

## Conclusion

This systematic review highlights that there are numerous PREMs available for people living with advanced serious illness, or their families globally. However, few directly align with what matters most to patients and families or are suitable for people with lower levels of literacy or with limited cognitive capacity due to illness. In taking this work forward, clinical teams firstly need to establish the core reason they want to collect and use PREM data a-priori. Secondly, administration of PREMs for this population is unlikely to be via a uniform approach and is likely to need personnel assistance to help patients in their completion of the questions. Who this person ought to be is unclear. However, careful planning to enable participation ensures the voice of this population is heard and informs service improvement work. Thirdly, mechanisms for data capture and timely feedback also need to be considered if these data are to inform the understanding of local needs, drive improvements and evaluate interventions.

## Supplemental Material

sj-pdf-1-pmj-10.1177_02692163231169319 – Supplemental material for Description of patient reported experience measures (PREMs) for hospitalised patients with palliative care needs and their families, and how these map to noted areas of importance for quality care: A systematic review Click here for additional data file. Supplemental material, sj-pdf-1-pmj-10.1177_02692163231169319 for Description of patient reported experience measures (PREMs) for hospitalised patients with palliative care needs and their families, and how these map to noted areas of importance for quality care: A systematic review by Claudia Virdun, Maja Garcia, Jane L Phillips and Tim Luckett in Palliative Medicine
