# Supplementary material for: Description of patient reported experience measures (PREMs) for hospitalised patients with palliative care needs and their families, and how these map to noted areas of importance for quality care: A systematic review
Source: Palliat Med. 2023 Apr 24;37(7):898–914. doi: 10.1177/02692163231169319 (PMC10320712; doi:10.1177/02692163231169319)
Supplement: sj-pdf-1-pmj-10.1177_02692163231169319 – Supplemental material for Description of patient reported experience measures (PREMs) for hospitalised patients with palliative care needs and their families, and how these map to noted areas of importance for quality care: A systematic review [file sj-pdf-1-pmj-10.1177_02692163231169319.pdf]

Supplementary Tables:

Table 1: MEDLINE search strategy

	**Searches**
1	(dying or death or 'end of life' or terminal or 'terminal care' or 'terminally ill' or palliative or 'final day*').mp. [mp=title, abstract, original title, name of substance word, subject heading word, floating sub-heading word, keyword heading word, organism supplementary concept word, protocol supplementary concept word, rare disease supplementary concept word, unique identifier, synonyms]
2	(patient* or family or families or consumer* or carer*).mp. [mp=title, abstract, original title, name of substance word, subject heading word, floating sub-heading word, keyword heading word, organism supplementary concept word, protocol supplementary concept word, rare disease supplementary concept word, unique identifier, synonyms]
3	Adult/ or adult*.mp.
4	((((Patient* or Consumer*) adj (satisfaction or Experience* or Opinion* or Perspective*)).m_titl. or exp Patient Satisfaction/) and ((Questionnaire* or Instrument* or measure*).m_titl. or (Health care surveys/ or questionnaires/))) or exp Patient Reported Outcome Measures/
5	1 and 2 and 3 and 4

Table 2: CINAHL search strategy

	**Search Options**
S14	S7 AND S11 AND S12 AND S13
S13	TX ((patient* or family or families or consumer* or carer*))
S12	TI ((Questionnaire* OR Instrument* OR measure*)) OR TX (("Health*care surveys" OR "health*care questionnaires")) OR MH patient reported outcome measures
S11	S8 OR S9 OR S10
S10	quality N3 care
S9	TX "patient satisfaction"
S8	TI (patient* OR consumer*) N (satisfaction OR experience* OR opinion* OR perspective*)
S7	S1 OR S2 OR S3 OR S4 OR S5 OR S6
S6	"final day*"
S5	"palliative" OR (MH "Palliative Care")
S4	"terminal care" OR (MH "Terminal Care+")
S3	"terminal" OR (MH "Terminally Ill Patients+")
S2	"end of life"
S1	"dying" OR (MH "Death+")

Table 3: PsycINFO search strategy

	**Search Options**
S14	S7 AND S11 AND S12 AND S13
S13	TX ((patient* or family or families or consumer* or carer*))
S12	TI ((Questionnaire* OR Instrument* OR measure*)) OR TX (("Health*care surveys" OR "health*care questionnaires")) OR MH patient reported outcome measures
S11	S8 OR S9 OR S10
S10	quality N3 care
S9	TX "patient satisfaction"
S8	TI (patient* OR consumer*) N (satisfaction OR experience* OR opinion* OR perspective*)
S7	S1 OR S2 OR S3 OR S4 OR S5 OR S6
S6	"final day*"
S5	"palliative" OR (MH "Palliative Care")
S4	"terminal care" OR (MH "Terminal Care+")
S3	"terminal" OR (MH "Terminally Ill Patients+")
S2	"end of life"
S1	"dying" OR (MH "Death+")

Supplementary Table 4: Review of reliability and validation measures applied to each included PREM

	Reliability		Validity				
PREM	**Reliability** *Test-retest; inter-rater; intra-rater*	**Internal consistency**	**Content validity** *Face validity*	**Construct validity**			**Responsiveness**
				Structural	*Cross-cultural*	*Hypothesis testing*	
consideRATE, 2021[1]* USA	Y	N	N	Y	N	Y	N
The Sinclair Compassion Questionnaire (SCQ), 2020[2] Canada	Y	Y	Y	Y	Y	Y	N
Quality Care Questionnaire-Palliative Care (QCQ-PC), 2018[3] Korea	N	Y	N	Y	N	N	N
Victorian Palliative Care Satisfaction Instrument (VPCSI),[4] 2016* Australia	Y	N	N	N	N	N	N
Quality from the Patient's Perspective (QPP-PC), 2015[5] Norway	N	Y	Y	Y	N	N	N
Quality of End-of-life Care and Satisfaction with Treatment (QUEST)[6] questionnaire, 2013	Y	Y	N	Y	N	N	N

	Reliability		Validity				
**PREM**	**Reliability** *Test-retest; inter-rater; intra-rater*	**Internal consistency**	**Content validity** *Face validity*	**Construct validity**			**Responsiveness**
				Structural	*Cross-cultural*	*Hypothesis testing*	
USA							
Satisfaction with treatment decision (SWTD) survey, 2013[7] Switzerland	N	Y	N	N	N	N	N
Consumer Quality Index Palliative Care questionnaire for patients, 2012, 2013[8] The Netherlands	N	N	Y	N	N	N	N
Quality of End-of-Life Care (QOELC) Survey – Patient, 2010[9] USA	N	N	N	Y	N	Y	N
FAMCARE-Patient, 2009[10] Australia	Y	Y	N	Y	N	Y	N
Quality of Communication Questionnaire (QOC), 2006[11] USA	N	Y	Y	Y	N	Y	N

	Reliability		Validity				Responsiveness
**PREM**	**Reliability** *Test-retest; inter-rater; intra-rater*	**Internal consistency**	**Content validity** *Face validity*	**Construct validity**			**Responsiveness**
				Structural	*Cross-cultural*	*Hypothesis testing*	
Palliative Care Quality of Life Instrument (PQLI), 2004[12] Cyprus	Y	Y	N	Y	N	Y	N
Kaiser Permanente Survey, 2020, 2001[13] USA	N	N	Y	N	N	Y	N
Otani et al. 2020 study developed questionnaire[14] Japan	N	N	Y	Y	N	N	N
Care of the Dying Evaluation (CODE) / international CODE (i-CODE), 2019[15] United Kingdom	N	Y	N	Y	N	N	N
Dying Care Outcome and Process Scale Before and After Death, 2019[16] Japan	Y	Y	N	Y	N	Y	N
Consumer Assessment of Healthcare Providers and Systems (CAHPS) Cancer Care Survey, 2017[17] USA	Y	Y	Y	Y	N	Y	N

	Reliability		Validity				Responsiveness
PREM	Reliability<br>*Test-retest; inter-rater; intra-rater*	Internal consistency	Content validity<br>*Face validity*	Construct validity			Responsiveness
				Structural	*Cross-cultural*	*Hypothesis testing*	
Caregiver Voice Survey, 2017[18]<br>Canada	Y	Y	Y	Y	Y	Y	N
euroQ2 Satisfaction with Care in the ICU, 2017[19]<br>Denmark	N	N	N	N	N	Y	N
Bereaved Family Survey (BFS), 2016[20]<br>USA	Y	N	N	Y	N	Y	N
Family Evaluation of Hospice Care (FEHC), 2015[21]<br>USA	N	N	N	Y	N	Y	N
Quality of Family Experience (QUAL-E Fam), 2014[22]<br>USA	Y	Y	N	Y	N	Y	N
Consumer Quality Index Palliative Care questionnaire for relatives, 2013[8]<br>The Netherlands	Y	N	N	Y	N	Y	N

	Reliability		Validity				Responsiveness
**PREM**	**Reliability** *Test-retest; inter-rater; intra-rater*	**Internal consistency**	**Content validity** *Face validity*	**Construct validity**			**Responsiveness**
				Structural	*Cross-cultural*	*Hypothesis testing*	
Canadian Health Care Evaluation Project (CANHELP) - Bereavement/Caregiver, 2010[23] Canada	N	Y	Y	Y	N	Y	Y
CANHELP Patient, 2010[23] Canada	N	Y	Y	Y	N	Y	Y
FAMCARE-2, 2010[24] Australia	N	Y	N	Y	N	Y	N
Quality of End-of-Life Care (QOELC) Survey – Family, 2010[9] USA	Y	N	N	Y	N	Y	N
Family Assessment of Treatment at the End-of-Life (FATE), 2008[25] USA	N	N	N	Y	N	Y	N
Family Satisfaction with Care in the Intensive Care Unit: FS-ICU 24R, 2007[26] Canada	Y	N	N	Y	N	Y	N

	Reliability		Validity				
**PREM**	**Reliability** *Test-retest; inter-rater; intra-rater*	**Internal consistency**	**Content validity** *Face validity*	**Construct validity**			**Responsiveness**
				Structural	*Cross-cultural*	*Hypothesis testing*	
CAHPS Hospice Survey, 2006[27] USA	N	Y	N	Y	N	Y	N
Care Evaluation Scale (CES), 2004[28] Japan	Y	N	N	Y	N	Y	N
Quality of Death and Dying Questionnaire (QODD), 2002[29] USA	N	Y	N	N	N	Y	N
Satisfaction Scale for Family Members Receiving Inpatient Palliative Care (SFIPC), 2002[30] Japan	Y	Y	N	N	N	Y	N
End-of-Life in Dementia Satisfaction With Care (SWC-EOLD), 2001[31] USA	Y	N	N	Y	N	Y	N
After death bereaved family member interview (hospital version) (Part of the Toolkit of Instruments to Measure End of Life Care [TIME]), 2000[32] USA	Y	Y	N	Y	N	Y	N
Primary Caregivers Satisfaction Survey, 1999[33] USA	N	N	Y	N	N	N	N

References

1. Saunders CH, Durand M-A, Scalia P, et al. User-Centered Design of the consideRATE Questions, a Measure of People's Experiences When They Are Seriously Ill. *Journal of Pain and Symptom Management* 2021; 61: 555-565.
2. Sinclair S, Jaggi P, Hack TF, et al. Initial validation of a patient-reported measure of compassion: Determining the content validity and clinical sensibility among patients living with a life-limiting and incurable illness. *The Patient: Patient-Centered Outcomes Research* 2020. DOI: 10.1007/s40271-020-00409-8.
3. Yun YH, Kang EK, Lee J, et al. Development and validation of the quality care questionnaire –palliative care (QCQ-PC): patient-reported assessment of quality of palliative care. *BMC Palliative Care* 2018; 17: 40. DOI: 10.1186/s12904-018-0296-2.
4. O'Connor M, Tan H and Lau R. Outcomes from applying a Palliative Care Satisfaction Survey Instrument in Victoria, Australia. *Progress in Palliative Care* 2016; 24: 93-97. DOI: 10.1179/1743291X15Y.0000000008.
5. Sandsdalen T, Rystedt I, Grøndahl VA, et al. Patients' perceptions of palliative care: adaptation of the Quality from the Patient's Perspective instrument for use in palliative care, and description of patients' perceptions of care received. *BMC palliative care* 2015; 14: 1-14.
6. Guerriere DN, Zagorski B and Coyte PC. Family caregiver satisfaction with home-based nursing and physician care over the palliative care trajectory: Results from a longitudinal survey questionnaire. *Palliative Medicine* 2013; 27: 632-638. DOI: 10.1177/0269216312473171.
7. Hitz F, Ribi K, Li Q, et al. Predictors of satisfaction with treatment decision, decision-making preferences, and main treatment goals in patients with advanced cancer. *Supportive Care in Cancer* 2013; 21: 3085-3093. DOI: https://dx.doi.org/10.1007/s00520-013-1886-4.
8. Claessen SJJ, Francke AL, Sixma HJ, et al. Measuring relatives' perspectives on the quality of palliative care: the consumer quality index palliative care. *Journal of Pain and Symptom Management* 2013; 45: 875-884.
9. Engelberg RA, Downey L, Wenrich MD, et al. Measuring the quality of end-of-life care. *Journal of Pain and Symptom Management* 2010; 39: 951-971.
10. Lo C, Burman D, Hales S, et al. The FAMCARE-Patient scale: Measuring satisfaction with care of outpatients with advanced cancer. *European Journal of Cancer* 2009; 45: 3182-3188. DOI: https://doi.org/10.1016/j.ejca.2009.09.003.
11. Engelberg R, Downey L and Curtis JR. Psychometric Characteristics of a Quality of Communication Questionnaire Assessing Communication about End-of-Life Care. *Journal of Palliative Medicine* 2006; 9: 1086-1098. DOI: 10.1089/jpm.2006.9.1086.
12. Mystakidou K, Tsilika E, Kouloulias V, et al. The 'Palliative Care Quality of Life Instrument (PQLI)' in terminal cancer patients. *Health and Quality of Life Outcomes* 2004; 2.
13. Glass DP, Wang SE, Minardi PM, et al. Concordance of end-of-life care with end-of-life wishes in an integrated health care system. *JAMA network open* 2021; 4: e213053-e213053.
14. Otani H, Morita T, Igarashi N, et al. A Nationwide Survey of Bereaved Family Members' Perception of the Place Patients Spent Their Final Days: Is the Inpatient Hospice Like or Unlike a Home? Why? *Palliative Medicine Reports* 2020; 1: 174-178.

15. Mayland CR, Gerlach C, Sigurdardottir K, et al. Assessing quality of care for the dying from the bereaved relatives' perspective: Using pre-testing survey methods across seven countries to develop an international outcome measure. *Palliative Medicine* 2019; 33: 357-368. DOI: 10.1177/0269216318818299.
16. Kanno Y, Sato K, Shimizu M, et al. Validity and reliability of the dying care process and outcome scales before and after death from the bereaved family members' perspective. *American Journal of Hospice and Palliative Medicine®* 2019; 36: 130-137.
17. Evensen CT, Yost KJ, Keller S, et al. Development and testing of the CAHPS cancer care survey. *Journal of oncology practice* 2019; 15: e969-e978.
18. Seow H, Bainbridge D, Brouwers M, et al. Validation of a modified VOICES survey to measure end-of-life care quality: the CaregiverVoice survey. *BMC Palliative Care* 2017; 16: 44.
19. Jensen HI, Gerritsen RT, Koopmans M, et al. Satisfaction with quality of ICU care for patients and families: the euroQ2 project. *Critical Care* 2017; 21: 1-10.
20. Thorpe JM, Smith D, Kuzla N, et al. Does mode of survey administration matter? Using measurement invariance to validate the mail and telephone versions of the bereaved family survey. *Journal of Pain and Symptom Management* 2016; 51: 546-556.
21. Holland JM, Keene JR, Kirkendall A, et al. Family evaluation of hospice care: Examining direct and indirect associations with overall satisfaction and caregiver confidence. *Palliative and Supportive Care* 2015; 13: 901-908. 2014/07/06. DOI: 10.1017/s1478951514000595.
22. Steinhauser KE, Voils CI, Bosworth HB, et al. Validation of a measure of family experience of patients with serious illness: The QUAL-E (Fam). *Journal of Pain and Symptom Management* 2014; 48: 1168-1181. DOI: 10.1016/j.jpainsymman.2014.04.006.
23. Heyland DK, Cook DJ, Rocker GM, et al. The development and validation of a novel questionnaire to measure patient and family satisfaction with end-of-life care: the Canadian Health Care Evaluation Project (CANHELP) Questionnaire. *Palliative Medicine* 2010; 24: 682-695. 2010/07/08. DOI: 10.1177/0269216310373168.
24. Aoun S, Kristjanson LJ, Oldham L, et al. A qualitative investigation of the palliative care needs of terminally ill people who live alone. *Collegian* 2008; 15: 3-9.
25. Casarett D, Pickard A, Bailey FA, et al. A Nationwide VA Palliative Care Quality Measure: The Family Assessment of Treatment at the End of Life. *Journal of Palliative Medicine* 2008; 11: 68-75. DOI: 10.1089/jpm.2007.0104.
26. Wall RJ, Engelberg RA, Downey L, et al. Refinement, scoring, and validation of the Family Satisfaction in the Intensive Care Unit (FS-ICU) survey. *Critical care medicine* 2007; 35: 271-279.
27. Crofton C, Lubalin JS and Darby C. Consumer Assessment of Health Plans Study (CAHPS). Foreword. *Medical Care* 1999; 37: MS1-MS9.
28. Morita T, Hirai K, Sakaguchi Y, et al. Measuring the quality of structure and process in end-of-life care from the bereaved family perspective. *Journal of Pain and Symptom Management* 2004; 27: 492-501.
29. Curtis JR, Patrick DL, Engelberg RA, et al. A measure of the quality of dying and death: Initial validation using after-death interviews with family members. *Journal of Pain and Symptom Management* 2002; 24: 17-31. DOI: 10.1016/S0885-3924(02)00419-0.
30. Morita T, Chihara S and Kashiwagi T. A scale to measure satisfaction of bereaved family receiving inpatient palliative care. *Palliative Medicine* 2002; 16: 141-150. DOI: 10.1191/0269216302pm514oa.

31. Volicer L, Hurley AC and Blasi ZV. Scales for evaluation of end-of-life care in dementia. *Alzheimer Disease and Associated Disorders* 2001; 15: 194-200.
32. Teno JM, Clarridge B, Casey V, et al. Validation of toolkit after-death bereaved family member interview. *Journal of Pain and Symptom Management* 2001; 22: 752-758.
33. Archer KC and Boyle DP. Toward a measure of caregiver satisfaction with hospice social services. *Hospice Journal* 1999; 14: 1-15. DOI: 10.1300/J011v14n02_01.
